# Comparison of Metabolic Control, Dietary Habits, Activity, and Psychological Condition in Children and Adolescents Treated with Personal Insulin Pumps

**DOI:** 10.3390/nu17203304

**Published:** 2025-10-21

**Authors:** Agnieszka Lejk, Karolina Myśliwiec, Jędrzej Chrzanowski, Jacek Burzyński, Arkadiusz Michalak, Malwina Musiał-Paździor, Marta Bandura, Jolanta Rutkowska-Kośmińska, Kinga Drzewińska, Aleksandra Grabowska, Mateusz Okonek, Marta Herstowska, Michał Hoffmann, Wojciech Fendler

**Affiliations:** 1 Clinic of Pediatrics, Diabetology and Endocrinology, University Clinical Center of Gdansk, 80-952 Gdansk, Polandj.u.rutkowska@gmail.com (J.R.-K.);; 2 Department of Pediatrics, Diabetology and Endocrinology, Medical University of Gdansk, 80-210 Gdansk, Poland; 3 Clinic of Arterial Hypertension and Diabetology, University Clinical Center of Gdansk, 80-952 Gdansk, Poland; 4 Department of Biostatistics and Translational Medicine, Medical University of Lodz, 92-215 Lodz, Polandjacek.burzynski@umed.lodz.pl (J.B.); arkadiusz.michalak@umed.lodz.pl (A.M.);

**Keywords:** insulin pump, eating habits, activity

## Abstract

Background: Type 1 diabetes mellitus (T1DM) is one of the most frequently occurring chronic metabolic conditions in the pediatric and adolescent population. That is why our aim in this study was to compare metabolic control, eating habits, activity, and mental health in patients using insulin pumps with predictive low glucose suspend (PLGS) and advanced hybrid closed loop (AHCL) systems. Methods: We selected 37 patients and collected clinical, continuous glucose monitoring (CGM), and question-naire data (food frequency questionnaire (FFQ-6), physical activity questionnaire for children (PAQ-C), pediatric quality of life inventory (PedsQL). Additionally, all pa-tients participated in culinary workshops, which included education on a low-glycemic-index diet. Results: We observed a significant difference between the PLGS and the AHCL groups for mean glucose, coefficient of variation, and Time in Range (≤54, 70–140, 70–180, ≥180, and ≥250 mg/dL). Patients with higher Time Below Range consumed juices or sugary drinks more frequently. All participants had incor-rect eating habits and engaged in irregular physical activity. Conclusions: We observed no significant differences in the diabetes-specific quality of life scores between the PLGS and AHCL groups.

## 1. Introduction

Type 1 diabetes mellitus (T1DM) is one of the most frequently occurring chronic metabolic conditions in the pediatric and adolescent population [1]. T1DM is an autoimmune disease characterized by the destruction of pancreatic β-cells, resulting in absolute insulin deficiency and impaired regulation of blood glucose levels [2]. Poor glycemic control is associated with long-term microvascular and macrovascular complications and a higher risk of mortality [3].

Epidemiological data indicate that more than 20,000 individuals aged 0–19 years in Poland are currently living with T1DM [4]. Over the past five years, the incidence in this age group has risen approximately 1.5-fold, reflecting a concerning upward trend [5].

In Poland, insulin pumps are a widely available therapeutic option for pediatric patients with T1DM. Patients may use pumps without predictive low glucose suspend (PLGS), pumps equipped with PLGS, or advanced hybrid closed-loop (AHCL) systems. PLGS-enabled pumps such as the Medtronic 640G and 740G (The producer: Medtronic MiniMed, Minneapolis, MN, USA) suspend insulin delivery when glucose levels fall and restart once they normalize, providing strong protection against hypoglycemia [6]. Advanced Hybrid Closed Loop (AHCL) systems are innovative forms of hybrid closed loop (HCL) insulin delivery systems, representing the latest development in automated insulin delivery (AID) technology. In pediatric populations, AHCL systems have been shown to improve metabolic control and restore hypoglycemia awareness [7].

Long-term management of T1DM requires lifelong subcutaneous insulin therapy combined with appropriate physical activity and a carefully planned diet, which together are essential components of efficacious disease treatment [8]. Many studies indicate that dietary habits and physical activity levels significantly influence metabolic control and, consequently, the risk of developing diabetes complications [9,10,11,12]. Metabolic control of diabetes may be defined as the degree to which metabolic parameters like blood glucose, glycated hemoglobin (HbA1c), lipid profile, body weight, and blood pressure are maintained within recommended target ranges. This concept is supported by evidence linking poor glycemic control with disrupted lipid metabolism and body weight dysregulation in pediatric T1DM populations [13].

Previous studies suggest that proper dietary habits, particularly a diet based on low-glycemic-index (GI) products, promote greater glycemic stability in children with T1DM and, consequently, better metabolic control [10]. Adequate intake of unprocessed vegetables is also considered an essential component of the diet in pediatric patients with T1DM and may facilitate improved glycemic regulation. Additionally, it is important to note that a well-balanced diet with a low glycemic index is a key component in optimizing pump therapy in T1DM. Proper eating habits, including maintaining regular meal times, selecting appropriate foods, avoiding simple carbohydrates, and eliminating highly processed foods, significantly contribute to proper metabolic control of the disease. Numerous scientific studies and International Society for Pediatric and Adolescent Diabetes (ISPAD) recommendations strongly emphasize that proper counting the grams of carbohydrates, assessment of the macronutrient composition of meals, and correct selection of products in accordance with the principles of a low-glycemic-index diet, also taking into account the glycemic load, affects the achievement of a higher time in range (TIR) regardless of the type of insulin pump used [11].

Physical activity has likewise been shown to play a crucial role in metabolic outcomes. Higher levels of physical activity in children and adolescents with T1DM are associated with better glycemic control, reflected by lower HbA1c levels. This association is even stronger for good cardiorespiratory fitness (CRF), which correlates with more favorable HbA1c outcomes [12]. Conversely, a sedentary lifestyle is associated with worse glycemic control, underscoring the importance of prevention and interventions that aim to increase physical activity and improve fitness as key components of therapeutic strategies in this patient population [12].

Psychological factors are also closely related to metabolic control in T1DM. In a large pediatric cohort, Albright et al. (2024) demonstrated that depressive symptoms and diabetes-related distress were associated with higher HbA1c, with diabetes distress fully mediating the relationship between depression and glycemic outcomes [14].

Given these findings, the present study aims to compare the effectiveness of glycemic control between AHCL and PLGS systems and to evaluate dietary habits and physical activity in patients using these devices. Furthermore, it seeks to determine whether dietary habits, physical activity, and quality of life differ according to the degree of metabolic control.

## 2. Materials and Methods

### 2.1. Participants, Recruitment and Study Design

We recruited pediatric patients with type 1 diabetes diagnosed according to the criteria of the ISPAD guidelines, remaining under the care of the Clinic of Pediatrics, Diabetology, and Endocrinology at the Medical University of Gdansk, Poland. From among these, we selected a total of N = 37 children using two categories of insulin pumps: Medtronic 640G or 740G with PLGS (N = 16) and Medtronic 780G (Medtronic MiniMed, Minneapolis, MN, USA) (N = 21) with an AHCL system. All data were collected from a continuous glucose monitoring system (CGM) and questionnaires regarding nutritional habits, physical activity, and mental state between July 2024–September 2024.

Additionally, all the patients participated in culinary workshops, which included education on a low-glycemic-index diet. In total, 5 culinary workshops of 2 h each were held over a period of 3 months (July–September). The entire therapeutic team was involved in organizing the workshops: a diabetologist, an educational nurse, a dietitian, and a psychologist. During the workshops, patients had the opportunity to receive advice and education on metabolic control, nutrition, physical activity, and improving their mental health. Patients had the opportunity to learn hands-on how to balance meals and choose appropriate foods. The dietitian taught about properly balanced meals and encouraged patients to choose whole grains. Additionally, they were retrained on counting carbohydrate grams in meals. Each patient received recipes for low-glycemic-index meals, which were also converted to carbohydrate grams. At the end of the workshop, there was a summary of current dietary recommendations for children and adolescents with type 1 diabetes.

### 2.2. Ethics Statement

The study protocol was approved by the Bioethical Commission of the Medical University of Gdansk (No. NKBBN/377/2024). Each participant and their parent were informed of the study protocol by the principal investigator and signed the written consent form.

### 2.3. Clinical and Continuous Glucose Monitoring Data

We collected clinical data such as body weight, height, duration of T1DM, duration of insulin pump therapy from the medical system. Additionally we used CGM systems (Guardian 3, Guardian 4) to measure glycemic variability proxy: daily mean glucose, coefficient of variation (CV), time below range (TBR ≤ 54 mg/dL and TBR ≤ 70 mg/dL), time above range (TAR ≥ 180 mg/dL and TAR ≥ 250 mg/dL), time in tight range (TITR = 70–140 mg/dL) and time in range (TIR = 70–180 mg/dL).

Patients’ CGM data were included in the analysis only if their CGM records satisfied the quality criteria, defined as at least 70% of daily sensor use for at least 14 days.

### 2.4. Food Preferences and Activity Data

Dietary habits were collected by the food frequency questionnaire FFQ-6. The Food Frequency Questionnaire (FFQ-6) was administered to patients under the close supervision of a dietitian. It is used to estimate usual eating habits. Patients had to choose 1 of 6 answers regarding their food consumption frequency in the last 12 months: (1) never or hardly ever, (2) once in a month or less frequently, (3) several times a month, (4) several times a week, (5) daily, (6) several times a day.

According to the questionnaire instructions, each question response was converted to a daily frequency (times/day) according to the following formula:

1—0; 2—0.025; 3—0.1; 4—0.571; 5—1.0; 6—2.0

Further analysis included 8 main food groups (sweets and snacks, dairy products and eggs, cereal products, fats, fruits, vegetables and grains, meat products and fish, beverages) and 3 additional groups: sweet drinks, simple sugars, and simple sugars without fruit. Daily frequency was calculated for each food group by summing the frequencies of the individual component foods.

We used the Modified Physical Activity Questionnaire (PAQ-C) to assess the level of physical activity among children and adolescents with Type 1 Diabetes Mellitus (T1DM). This questionnaire contains 10 questions about the various types of physical activity children engage in during the week. The questions also address physical activity at school, its frequency, and intensity. The PAQ-C was analyzed according to the instructions. The mean value for leisure-time physical activity (the mean frequency of the patient’s individual activities—Part 1 of the questionnaire) and the mean value for total physical activity (the mean frequency of Part 1 and Parts 2–9 of the questionnaire) were reported.

### 2.5. Psychological Data

Mental health was assessed using the Pediatric Quality of Life (PedsQL) for diabetes patients. It consists of 28 questions, each of which requires a selection based on the occurrence of a problem in the past month, divided into five sections: diabetes symptoms, barriers to treatment, treatment adherence, concerns about treatment effectiveness, and communication. Both patients’ and parents’ mental health was assessed, and the PedsQL questionnaire was analyzed according to guidelines.

### 2.6. Statistical Analysis

Anthropometric data were converted to z-scores and percentiles using the OLAF and OLA percentile charts. Nominal variables were described using counts and percentages, and continuous variables were described using the median and the lower and upper quartiles. Comparisons of continuous variables between two unpaired groups were performed using the nonparametric Mann–Whitney U test. Spearman’s rank correlation was used to assess the relationship between two continuous variables. Statistically significant relationships were presented using a scatterplot. Analysis was performed using R (v4.4.1). CGM recordings were analyzed using GlyCulator 3.0. The threshold for statistical significance was α = 0.05. Multiple testing adjustment was done with the Benjamini–Hochberg method, with adjusted values provided in Supplementary Tables.

## 3. Results

### 3.1. Characteristics of the Study Group

In total, we recruited 37 patients, including 16 (43.2%) females and 21 (56.8%) males. All patients were Caucasian, and on clinical evaluation reported only T1DM, with no signs of malnutrition. Twenty-one patients (56.8%) used a hybrid closed-loop (HCL; Medtronic 780G) system, while 16 patients (43.2%) were treated with Predictive Low Glucose Suspend (PLGS) technology. No significant differences between the HCL and PLGS groups were observed regarding to age and anthropometric data. The detailed group characteristics are provided in Table 1.

**Figure 1:**
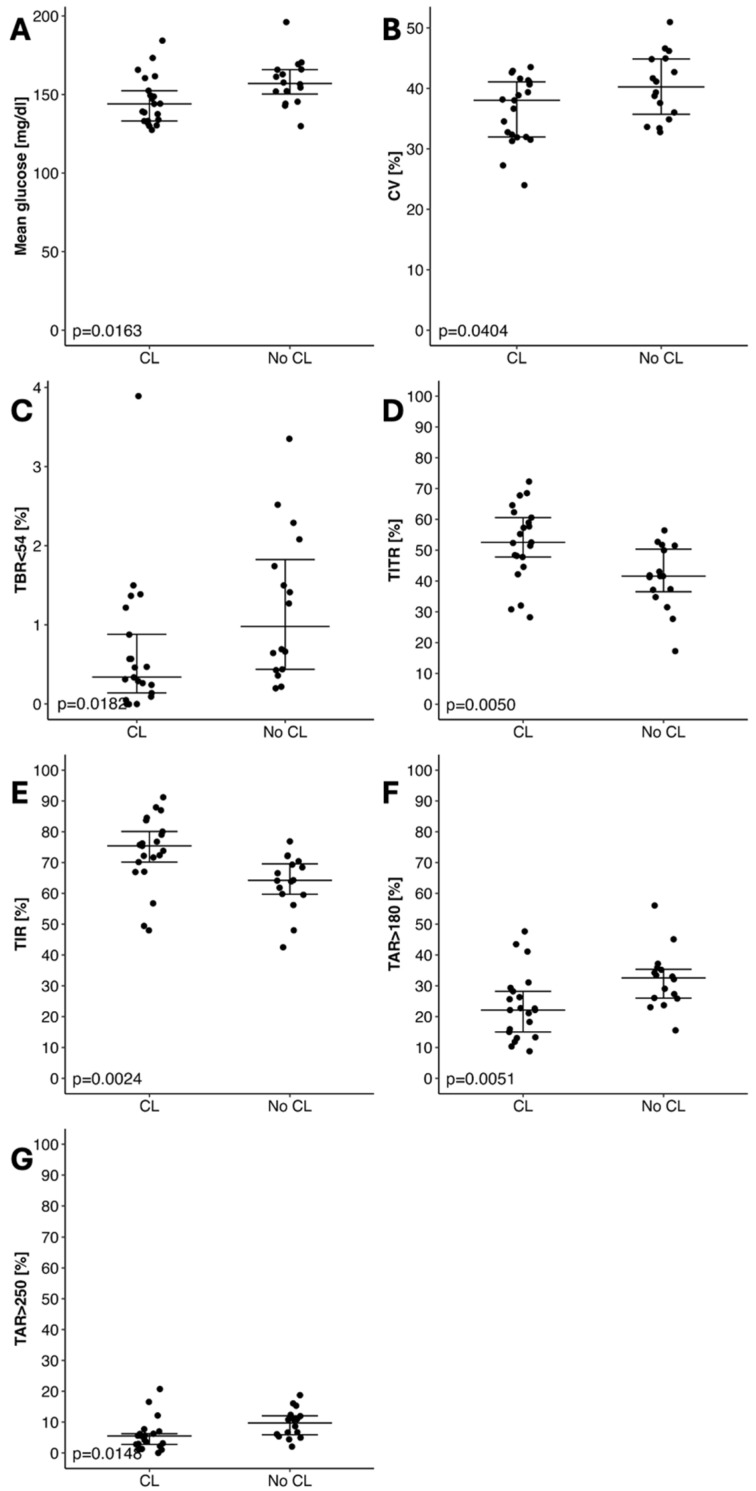
Comparison between particular glycemic variables and the model of insulin pump: (**A**) Mean glucose (mg/dL); (**B**) Coefficient of Variation [%], (**C**) Time Below Range ≤ 54 mg/dL; (**D**) Time in Tight Range (%); (**E**) Time in Range (%); (**F**) Time Above Range ≥ 180 mg/dL; (**G**) Time Above Range ≥ 250 mg/dL.

As expected, patients using the HCL system achieved better glycemic control compared to those on PLGS (Table 2, Figure 1). We did not observe a significant difference regarding the quality of life, physical activity, and dietary habits between study groups (Table S1).

### 3.2. Association Between Glycemic Variability and Eating Habits, Activity, and Mental Health

**Figure 2:**
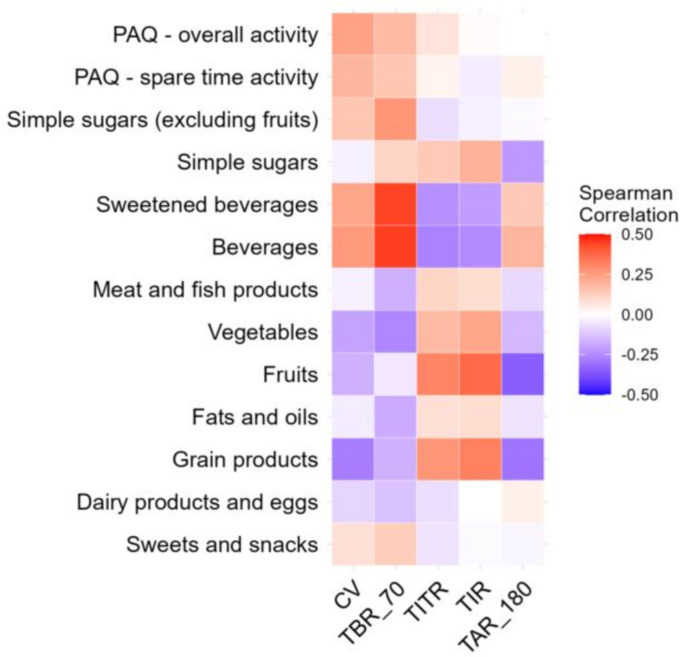
Spearman Correlation between glycemic variability FFQ-6 and PAQ.

Comparing metabolic control of the disease, dietary habits, physical activity level, and mental health, we noticed that patients who spent more time in hypoglycemia (higher Time Below Range) consumed juices or sugary drinks more frequently. Figure 2 shows this relationship using the Spearman correlation. According to FFQ-6 data, patients in both analyzed groups did not follow the nutritional recommendations, and dietary errors were comparable, characterized by a high daily intake of carbohydrates compared to the amounts of fat and protein, as well as a tendency to reach for sweet snacks or sweet drinks. The observed differences in the consumption of various dietary products between the mentioned groups were mostly not significant. Additionally, the PAQ-C survey revealed that patients do not engage in regular physical activity either at school or outside of school. They also rarely participate in additional physical activities. There were also no significant differences between the groups in terms of mental health.

**Figure 3:**
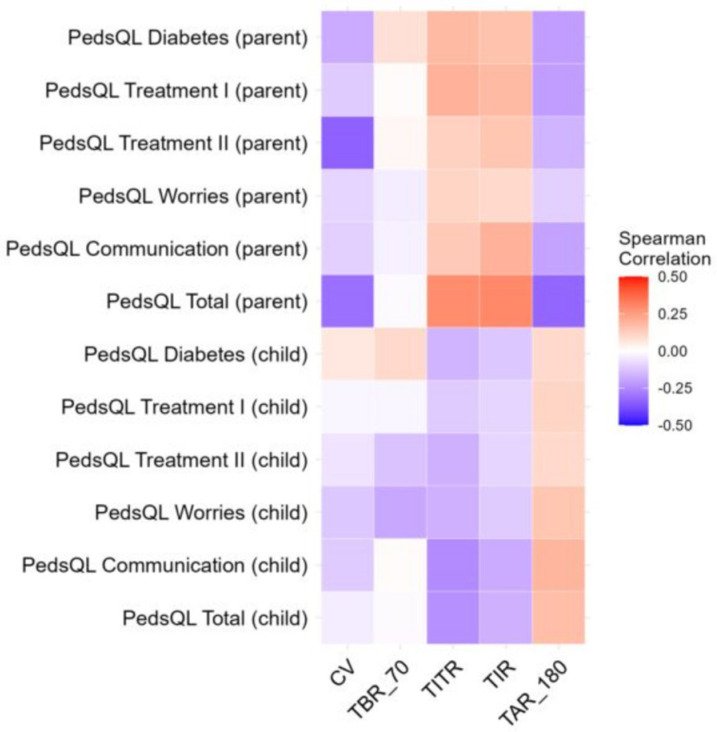
Spearman Correlation between glycemic variability and PedsQL.

No other significant correlations were found between dietary habits, physical activity level, mental health, and metabolic control of the disease (Figure 3). The detailed results of correlations are provided in Table S2.

## 4. Discussion

The results of our study lead to some interesting conclusions. Firstly, we found that glycemic control significantly differs between patients treated with the PLGS and the AHCL system. The usage of AHCL is associated with a higher percentage of TIR compared to the patients treated with the PLGS system. Moreover, the difference in time in tight range (TITR) between the groups was also statistically significant. These statements are consistent with the results of many other studies comparing metabolic control between patients treated with different insulin delivery systems [15,16,17,18]. The large meta-analysis performed by Michou et al. highlighted the superiority of automated insulin delivery systems among children and adolescents [19]. Our results show that this is the crucial factor determining diabetes control among the pediatric population.

Additionally, in our analysis, patients using AHCLs were more frequently achieving the targeted value of coefficient of variation (CV), a clinically important indicator for glucose variability [20]. This indicates improved metabolic control and a reduced risk of both hypoglycemia and severe hyperglycemia. Therefore, such patients are more likely to avoid many acute or chronic complications related to diabetes mellitus type 1 (T1DM).

Regardless of the type of insulin delivery system, nutritional choices play a crucial role in diabetes management. According to FFQ-6 data, patients in both analyzed groups did not follow the nutritional recommendations. These results are consistent with other studies, which show that awareness of healthy nutrition could be improved [21]. Our findings should be interpreted with caution, as the dietary evaluation relied solely on self-reported FFQ-6 data and did not include baseline measurements or detailed nutritional assessments (e.g., food diaries, biomarkers, nutrient analysis) and lack objective validation, which limits the strength of nutritional conclusions. The proper education of pediatric patients and their families not only ameliorates the metabolic control of T1DM but also improves their overall quality of life. The study performed by Dłużniak-Gołaska et al. showed that nutritional education, regardless of the method, is a crucial factor influencing different aspects of the quality of life in such patients [22].

Numerous publications emphasize the importance of a well-balanced diet to optimize pump therapy in children and adolescents with type 1 diabetes. Appropriate carbohydrate gram counting, a low-glycemic-index diet, including the right selection of foods, and regular meals significantly improve metabolic control of the disease, regardless of the type of insulin pump used. It is important to emphasize that nutrition is a key component of type 1 diabetes treatment [23,24]. However, in our study, patients lacked healthy eating habits, which also impacted their metabolic control and decreased time in the target range.

The adequate amount of daily physical activity recommended by ISPAD [25] for children with T1DM proved difficult to achieve for our study participants. Our results support the notion that following everyday requirements is a challenge for children and adolescents with T1DM [26]. Due to the limited data available, future research comparing participants’ metabolic control before and after increasing the amount of time spent on exercise may facilitate a better assessment and understanding of the role of physical activity in diabetes management.

The level of glycemic control may, to some extent, influence the nutritional choices made by individuals with T1DM. We observed that patients with higher time spent in hypoglycemia (TBR < 70 mg/dL) were more likely to drink sweet beverages. However, this behavior may result from the fact that incidents of hypoglycemia are more likely to occur in patients treated with the PLGS system, whose metabolic control was worse, compared to those with AHCLs. It is also possible that patients consume sweet beverages as a way to manage low blood glucose; however, hypoglycemia itself may also result from other factors, such as administering excessive insulin doses, improper dietary habits, or inaccurate carbohydrate counting.

### Limitations

Limitations of this study must be acknowledged. The small sample size (N = 37) limits the statistical power and generalizability of the results. Participants were assessed at a single time point; no baseline HbA1c or pre-pump glycemic data were collected, making it difficult to confirm the comparability of groups at study entry. The 3-month observation window is insufficient to evaluate long-term metabolic or behavioral changes. Confounding factors (such as socioeconomic status, parental education, and prior diabetes education) and other potential sources of systematic bias were not assessed. Dietary habits and physical activity were assessed using self-reported FFQ-6 and PAQ-C questionnaires, both of which are prone to recall and social desirability bias. In particular, the nutritional assessment was limited to questionnaire data and workshop participation, without incorporating biomarkers or quantitative dietary outcomes, which restricts the integration of dietary findings with metabolic results. Future studies utilizing objective measures, such as accelerometers or food diaries, are needed to validate the observed results. Finally, all participants received standardized dietary education through culinary workshops, which may have reduced observable differences between groups.

## 5. Conclusions

In conclusion, our study confirmed that AHCLs provide better metabolic control than PLGS systems among pediatric patients with T1DM. Following the recommendations on the appropriate nutritional habits and physical activity remains challenging.

## Figures and Tables

**Table 1:** Characteristics of the Study Group.

Variable	HCL (N = 21)	PLGS (N = 16)	*p* Value
Age [years]	10.42 (9.13–12.13)	9.78 (9.07–11.84)	0.5959
Weight [kg]	37 (31.2–43.2)	34.45 (29.25–38.5)	0.3576
Weight [z-score]	−0.08 (−1.1–0.78)	−0.22 (−0.77–0.49)	0.5750
Weight [percentile]	46.89 (13.59–78.21)	41.25 (22.64–68.76)	-
Height [cm]	144.8 (136.4–151.1)	133.8 (131–148.5)	0.2761
Height [z-score]	0.11 (−0.88–0.67)	−0.49 (−0.74–0.04)	0.2379
Height [percentile]	54.31 (19.03–74.88)	31.17 (23–51.6)	-
BMI [kg/m^2^]	16.89 (15.85–19.3)	17.64 (15.73–18.82)	0.8903
BMI [z-score]	−0.25 (−0.61–0.56)	0.29 (−0.72–0.56)	0.9879
BMI [percentile]	39.99 (27.03–71.37)	61.36 (23.88–71.31)	-
Disease duration [years]	4.07 (2.8–6.31)	3.34 (2.88–5.99)	>0.9999
DDI [U/kg/day]	0.73 (0.68–0.98)	0.78 (0.67–0.83)	0.9879
Basal insulin [%]	33 (28–40)	35 (27.75–38)	0.7872

DDI—Daily Dose of Insulin.

**Table 2:** Comparison of glycemic variability parameters.

Variable	HCL (N = 21)	PLGS (N = 16)	*p*
Mean glucose [mg/dL]	144.02 (133.17–152.43)	157.06 (150.28–165.79)	0.0193
CV (%)	38.03 (31.96–41.07)	40.24 (35.73–44.86)	0.0404
Time Below Target Range ≤ 54 mg/dL [%]	0.34 (0.14–0.88)	0.98 (0.44–1.82)	0.0182
Time in Tight Range 70–140 mg/dL [%]	52.52 (47.78–60.56)	41.57 (36.53–50.33)	0.0050
Time in Target Range 70–180 mg/dL [%]	75.4 (70.16–80.06)	64.2 (59.72–69.59)	0.0024
Time Above Range ≥ 180 mg/dL [%]	22.12 (15.02–28.22)	32.55 (25.98–35.34)	0.0051
Time Above Target Range ≥ 250 mg/dL [%]	5.51 (2.8–6.23)	9.74 (5.9–12.05)	0.0148

## Data Availability

The data presented in this study are available on request from the corresponding author. The data are not publicly available due to subjects privacy.

## Supplementary Materials

The following supporting information can be downloaded at: https://www.mdpi.com/article/10.3390/nu17203304/s1, Table S1. Characteristics of dietary habits, physical activity and quality of life; Table S2. Correlation between glycemic control and physical activity, dietary habits and quality of life.

## References

[1] Cockcroft E.J., Clarke R., Dias R.P., Lloyd J., Mann R.H., Narendran P., Reburn C., Smith B., Smith J.R., Andrews R.C. (2024). Effectiveness of Educational and Psychoeducational Self-Management Interventions in Children and Adolescents with Type 1 Diabetes: A Systematic Review and Meta-Analysis. Pediatr. Diabetes.

[2] Godoi A., Marques I.R., Padrão E.M.H., Mahesh A., Hespanhol L.C., Júnior J.E.R.L., de Souza I.A.F., Moreira V.C.S., Silva C.H., Miyawaki I.A. (2023). Glucose control and psychosocial outcomes with use of automated insulin delivery for 12 to 96 weeks in type 1 diabetes: A meta-analysis of randomised controlled trials. Diabetol. Metab. Syndr..

[3] Grabia M., Markiewicz-Żukowska R., Socha K. (2021). Prevalence of Metabolic Syndrome in Children and Adolescents with Type 1 Diabetes Mellitus and Possibilities of Prevention and Treatment: A Systematic Review. Nutrients.

[4] International Diabetes Federation (2019). IDF Diabetes Atlas. https://www.diabetesatlas.org.

[5] Szalecki M., Wysocka-Mincewicz M., Ramotowska A., Mazur A., Lisowicz L., Beń-Skowronek I., Sieniawska J., Klonowska B., Charemska D., Nawrotek J. (2018). Epidemiology of type 1 diabetes in Polish children: A multicentre cohort study. Diabetes Metab. Res. Rev..

[6] Adolfsson P., Hanas R., Zaharieva D.P., Dovc K., Jendle J. (2024). Automated Insulin Delivery Systems in Pediatric Type 1 Diabetes: A Narrative Review. J. Diabetes Sci. Technol..

[7] Schiaffini R., Deodati A., Nicoletti M.C., Carducci C., Ciampalini P., Lorubbio A., Matteoli M.C., Pampanini V., Patera I.P., Rapini N. (2022). Comparison of two advanced hybrid closed loop in a pediatric population with type 1 diabetes: A real-life observational study. Acta Diabetol..

[8] Jendle J.H., Riddell M.C. (2019). Editorial: Physical Activity and Type 1 Diabetes. Front. Endocrinol..

[9] Najafipour F., Mobasseri M., Yavari A., Nadrian H., Aliasgarzedeh A., Abbasi N.M., Niafar M., Gharamaleki J.H., Sadra V. (2017). Effect of regular exercise training on changes in HbA1c, BMI and VO2max among patients with type 2 diabetes mellitus: An 8-year trial. BMJ Open Diabetes Res. Care.

[10] Quarta A., Guarino M., Tripodi R., Giannini C., Chiarelli F., Blasetti A. (2023). Diet and Glycemic Index in Children with Type 1 Diabetes. Nutrients.

[11] Tayyem R., Zakarneh S., Al-Jayyousi G.F. (2023). Investigating the association between dietary patterns and glycemic control among children and adolescents with T1DM. Open Life Sci..

[12] Huerta-Uribe N., Ramírez-Vélez R., Izquierdo M., García-Hermoso A. (2023). Association Between Physical Activity, Sedentary Behavior and Physical Fitness and Glycated Hemoglobin in Youth with Type 1 Diabetes: A Systematic Review and Meta-analysis. Sports Med..

[13] Canha M., Ferreira S., Santos Silva R., Azevedo A., Rodrigues A.S., Castro-Correia C. (2023). Glycemic Control and Metabolic Parameters in Children and Adolescents with Type 1 Diabetes. Cureus.

[14] Albright D., Wardell J., Harrison A., Mizokami-Stout K., Hirschfeld E., Garrity A., Thomas I., Lee J. (2024). Screening for diabetes distress and depression in routine clinical care for youth with type 1 diabetes. J. Pediatr. Psychol..

[15] Collyns O.J., Meier R.A., Betts Z.L., Chan D.S., Frampton C., Frewen C.M., Hewapathirana N.M., Jones S.D., Roy A., Grosman B. (2021). Improved glycemic outcomes with medtronic minimed advanced hybrid closed-loop delivery: Results from a randomized crossover trial comparing automated insulin delivery with predictive low glucose suspend in peoplewithtype1diabetes. Diabetes Care.

[16] Burnside M.J., Lewis D.M., Crocket H.R., Meier R.A., Williman J.A., Sanders O.J., Jefferies C.A., Faherty A.M., Paul R.G., Lever C.S. (2023). Extended Use of an Open-Source Automated Insulin Delivery System in Children and Adults with Type 1 Diabetes: The 24-Week Continuation Phase Following the CREATE Randomized Controlled Trial. Diabetes Technol. Ther..

[17] Isganaitis E., Raghinaru D., Ambler-Osborn L., Pinsker J.E., Buckingham B.A., Wadwa R.P., Ekhlaspour L., Kudva Y.C., Levy C., Forlenza G.P. (2021). Closed-Loop Insulin Therapy Improves Glycemic Control in Adolescents and Young Adults: Outcomes from the International Diabetes Closed-Loop Trial. Diabetes Technol. Ther..

[18] Lejk A., Myśliwiec K., Michalak A., Pernak B., Fendler W., Myśliwiec M. (2024). Comparison of Metabolic Control in Children and Adolescents Treated with Insulin Pumps. Children.

[19] Michou P., Gkiourtzis N., Christoforidis A., Kotanidou E.P., Galli-Tsinopoulou A. (2023). The efficacy of automated insulin delivery systems in children and adolescents with type 1 diabetes Mellitus: A systematic review and meta-analysis of randomized controlled trials. Diabetes Res. Clin. Pract..

[20] Urakami T., Terada H., Tanabe S., Mine Y., Aoki M., Aoki R., Suzuki J., Morioka I. (2024). Clinical significance of coefficient of variation in continuous glucose monitoring for glycemic management in children and adolescents with type 1 diabetes. J. Diabetes Investig..

[21] Gitsi E., Livadas S., Angelopoulos N., Paparodis R.D., Raftopoulou M., Argyrakopoulou G. (2023). A Nutritional Approach to Optimizing Pump Therapy in Type 1 Diabetes Mellitus. Nutrients.

[22] Dłużniak-Gołaska K., Panczyk M., Szypowska A., Sińska B., Szostak-Węgierek D. (2020). Influence of two different methods of nutrition education on the quality of life in children and adolescents with type 1 diabetes mellitus—A randomized study. Rocz. Panstw. Zakl. Hig./Ann. Natl. Inst. Hyg..

[23] Leelarathna L., Choudhary P., Wilmot E.G., Lumb A., Street T., Kar P., Ng S.M. (2021). Hybrid Closed-loop Therapy: Where Are We in 2021?. Diabetes Obes. Metab..

[24] Breton M.D., Kanapka L.G., Beck R.W., Ekhlaspour L., Forlenza G.P., Cengiz E., Schoelwer M., Ruedy K.J., Jost E., Carria L. (2020). A Randomized Trial of Closed-Loop Control in Children with Type 1 Diabetes. N. Engl. J. Med..

[25] Adolfsson P., Taplin C.E., Zaharieva D.P., Pemberton J., Davis E.A., Riddell M.C., McGavock J., Moser O., Szadkowska A., Lopez P. (2022). ISPAD Clinical Practice Consensus Guidelines 2022: Exercise in children and adolescents with diabetes. Pediatr. Diabetes.

[26] Clerc A. (2023). Nutrition education to type 1 diabetes patients: Few changes over the time. Front. Clin. Diabetes Healthc..

