# Supplementary material for: Comparison of Metabolic Control, Dietary Habits, Activity, and Psychological Condition in Children and Adolescents Treated with Personal Insulin Pumps
Source: Nutrients. 2025 Oct 21;17(20):3304. doi: 10.3390/nu17203304 (PMC12566938; doi:10.3390/nu17203304)
Supplement: Supplementary file 1 [file nutrients-17-03304-s001.zip › nutrients-3873811-supplementary.pdf]

Table S1. Characteristics of dietary habits, physical activity and quality of life.

	Category	HCL (N=21)	PLGS (N=16)	p-value
Dietary habits (FFQ-6)	Sweet and snacks	0.9 (0.38 - 1.7)	1.34 (0.88 - 1.96)	0.2027
	Dairy products and eggs	1.86 (0.87 - 2.24)	2.48 (1.79 - 3.47)	0.0355
	Grain products	1.8 (1.12 - 2.24)	1.52 (1.07 - 2.34)	0.7942
	Fats and oils	1.87 (1.1 - 2.67)	1.78 (1.14 - 2.61)	0.9267
	Fruits	2.24 (1.57 - 2.84)	1.93 (1.48 - 3.37)	0.6565
	Vegetables	3.67 (2.52 - 4.95)	3.7 (2.4 - 4.88)	0.9633
	Meat and fish products	1.91 (1.34 - 2.58)	2.07 (0.96 - 2.36)	0.8781
	Beverages	0.07 (0.03 - 0.67)	0.22 (0.07 - 0.77)	0.3244
	Sweetened beverages	0.07 (0 - 0.57)	0.16 (0.04 - 0.75)	0.3010
	Simple sugars	3.88 (3.51 - 6.31)	5.05 (3.53 - 7.61)	0.4806
	Simple sugars (excluding fruits)	1.57 (1.27 - 3.9)	2.96 (1.92 - 4)	0.1676
Physical activity (PAQ)	Spare time activity	1.64 (1.45 - 2)	1.61 (1.44 - 1.96)	0.7819
	Total activity	2.62 (2.42 - 3.03)	2.65 (2.54 - 3.07)	0.5600
Quality of life (PedsQL)	Diabetes	0.41 (0.34 - 0.48)	0.39 (0.25 - 0.48)	0.5079
	Treatment I	0.38 (0.25 - 0.5)	0.38 (0.28 - 0.5)	0.5968
	Treatment II	0.18 (0.11 - 0.32)	0.12 (0.11 - 0.2)	0.3493
	Worries	0.42 (0.25 - 0.58)	0.42 (0.29 - 0.44)	0.8641
	Communication	0.33 (0.17 - 0.58)	0.25 (0.25 - 0.44)	0.7676
	Total	0.36 (0.3 - 0.41)	0.3 (0.25 - 0.36)	0.2191

PedsQL – Pediatric Quality of Life Inventory, **FFQ-6** – Food Frequency Questionnaire-6, **PAQ** – Physical Activity Questionnaire

Table S2. Correlation between glycemic control and physical activity, dietary habits and quality of life.

Correlation	R	p value	FDR
DDI & Sweets and snacks	0.10	0.5466	0.9126
DDI & Dairy products and eggs	0.11	0.5061	0.9126
DDI & Grain products	-0.16	0.3451	0.9126
DDI & Fats and oils	-0.03	0.8428	0.9126
DDI & Fruits	0.07	0.6758	0.9126
DDI & Vegetables	0.31	0.0656	0.9126
DDI & Meat and fish products	-0.12	0.4689	0.9126
DDI & Beverages	0.04	0.8305	0.9126
DDI & Sweetened beverages	-0.02	0.9126	0.9126
DDI & Simple sugars	0.06	0.7300	0.9126
DDI & Simple sugars (excluding fruits)	0.06	0.7285	0.9126
DDI & PAQ - spare time activity	-0.11	0.5173	0.9126
DDI & PAQ - overall activity	-0.04	0.7951	0.9126
DDI & Ankieta_sumarycznie	-0.19	0.2637	0.9126
DDI & PedsQL Diabetes	0.11	0.533	0.9126
DDI & PedsQL Treatment I	-0.07	0.6631	0.9126

DDI & PedsQL Treatment II	-0.14	0.3964	0.9126
DDI & PedsQL Worries	0.07	0.6903	0.9126
DDI & PedsQL Communication	0.02	0.9086	0.9126
DDI & PedsQL Total	-0.04	0.8231	0.9126
GMI & Sweets and snacks	0	0.9869	0.9869
GMI & Dairy products and eggs	-0.07	0.6945	0.8351
GMI & Grain products	-0.32	0.0511	0.3724
GMI & Fats and oils	-0.16	0.3555	0.6602
GMI & Fruits	-0.32	0.0529	0.3724
GMI & Vegetables	-0.2	0.2381	0.6131
GMI & Meat and fish products	-0.1	0.5636	0.8141
GMI & Beverages	0.01	0.9576	0.9869
GMI & Sweetened beverages	0.01	0.9441	0.9869
GMI & Simple sugars	-0.22	0.1972	0.6131
GMI & Simple sugars (excluding fruits)	-0.03	0.8497	0.9605
GMI & PAQ - spare time activity	-0.14	0.412	0.6695
GMI & PAQ - overall activity	0.1	0.5393	0.8141
GMI & Ankieta_sumarycznie	-0.15	0.3835	0.6647
GMI & PedsQL Diabetes	0.2	0.2426	0.6131
GMI & PedsQL Treatment I	0.24	0.1588	0.6131
GMI & PedsQL Treatment II	0.21	0.2022	0.6131
GMI & PedsQL Worries	0.32	0.0512	0.3724
GMI & PedsQL Communication	0.19	0.2594	0.6131
GMI & PedsQL Total	0.32	0.0573	0.3724
CV & Sweets and snacks	0.08	0.6245	0.8546
CV & Dairy products and eggs	-0.09	0.5838	0.8546
CV & Grain products	-0.28	0.0986	0.7601
CV & Fats and oils	-0.04	0.8279	0.8962
CV & Fruits	-0.17	0.3044	0.7914
CV & Vegetables	-0.2	0.2375	0.7914
CV & Meat and fish products	-0.03	0.8415	0.8962
CV & Beverages	0.26	0.1276	0.7601
CV & Sweetened beverages	0.23	0.1754	0.7601
CV & Simple sugars	-0.03	0.8617	0.8962
CV & Simple sugars (excluding fruits)	0.15	0.3598	0.8504
CV & PAQ - spare time activity	0.19	0.2629	0.7914
CV & PAQ - overall activity	0.24	0.1583	0.7601
CV & Ankieta_sumarycznie	-0.11	0.5124	0.8546
CV & PedsQL Diabetes	0.06	0.7418	0.8962
CV & PedsQL Treatment I	-0.02	0.9205	0.9205
CV & PedsQL Treatment II	-0.06	0.7136	0.8962
CV & PedsQL Worries	-0.12	0.4743	0.8546
CV & PedsQL Communication	-0.11	0.5142	0.8546
CV & PedsQL Total	-0.04	0.8191	0.8962
TBR_70 & Sweets and snacks	0.13	0.4349	0.8404

TBR_70 & Dairy products and eggs	-0.13	0.4525	0.8404
TBR_70 & Grain products	-0.17	0.3173	0.839
TBR_70 & Fats and oils	-0.18	0.299	0.839
TBR_70 & Fruits	-0.05	0.7465	0.9631
TBR_70 & Vegetables	-0.26	0.1216	0.702
TBR_70 & Meat and fish products	-0.17	0.3227	0.839
TBR_70 & Beverages	0.45	0.0056	0.0767
TBR_70 & Sweetened beverages	0.44	0.0059	0.0767
TBR_70 & Simple sugars	0.11	0.5254	0.8873
TBR_70 & Simple sugars (excluding fruits)	0.27	0.1057	0.702
TBR_70 & PAQ - spare time activity	0.15	0.3798	0.8404
TBR_70 & PAQ - overall activity	0.18	0.2774	0.839
TBR_70 & Ankieta_sumarycznie	-0.25	0.135	0.702
TBR_70 & PedsQL Diabetes	0.1	0.546	0.8873
TBR_70 & PedsQL Treatment I	-0.02	0.8899	0.9631
TBR_70 & PedsQL Treatment II	-0.13	0.4515	0.8404
TBR_70 & PedsQL Worries	-0.19	0.2603	0.839
TBR_70 & PedsQL Communication	0.01	0.9569	0.9631
TBR_70 & PedsQL Total	-0.01	0.9391	0.9631
TITR & Sweets and snacks	-0.06	0.7327	0.762
TITR & Dairy products and eggs	-0.07	0.6776	0.7604
TITR & Grain products	0.27	0.107	0.5497
TITR & Fats and oils	0.08	0.6434	0.7604
TITR & Fruits	0.31	0.0617	0.5497
TITR & Vegetables	0.18	0.2864	0.665
TITR & Meat and fish products	0.11	0.5202	0.7307
TITR & Beverages	-0.27	0.1107	0.5497
TITR & Sweetened beverages	-0.24	0.148	0.5497
TITR & Simple sugars	0.14	0.4128	0.7155
TITR & Simple sugars (excluding fruits)	-0.07	0.7019	0.7604
TITR & PAQ - spare time activity	0.03	0.8454	0.8454
TITR & PAQ - overall activity	0.07	0.6906	0.7604
TITR & Ankieta_sumarycznie	0.09	0.5995	0.7604
TITR & PedsQL Diabetes	-0.16	0.3325	0.665
TITR & PedsQL Treatment I	-0.11	0.534	0.7307
TITR & PedsQL Treatment II	-0.17	0.3103	0.665
TITR & PedsQL Worries	-0.17	0.3259	0.665
TITR & PedsQL Communication	-0.25	0.1416	0.5497
TITR & PedsQL Total	-0.24	0.1446	0.5497
TIR & Sweets and snacks	-0.01	0.9694	0.9789
TIR & Dairy products and eggs	0	0.9789	0.9789
TIR & Grain products	0.32	0.0533	0.6413
TIR & Fats and oils	0.09	0.5928	0.7994
TIR & Fruits	0.37	0.0261	0.6413
TIR & Vegetables	0.23	0.1747	0.7189

TIR & Meat and fish products	0.09	0.5763	0.7994
TIR & Beverages	-0.25	0.1405	0.7189
TIR & Sweetened beverages	-0.21	0.2154	0.7189
TIR & Simple sugars	0.2	0.2473	0.7189
TIR & Simple sugars (excluding fruits)	-0.03	0.8672	0.9789
TIR & PAQ - spare time activity	-0.04	0.7935	0.9789
TIR & PAQ - overall activity	0.01	0.9533	0.9789
TIR & Ankieta_sumarycznie	0.03	0.8522	0.9789
TIR & PedsQL Diabetes	-0.12	0.4754	0.7994
TIR & PedsQL Treatment I	-0.09	0.6149	0.7994
TIR & PedsQL Treatment II	-0.09	0.5964	0.7994
TIR & PedsQL Worries	-0.11	0.5139	0.7994
TIR & PedsQL Communication	-0.18	0.2777	0.7189
TIR & PedsQL Total	-0.17	0.318	0.7189
TAR_180 & Sweets and snacks	-0.02	0.9156	0.9817
TAR_180 & Dairy products and eggs	0.04	0.8359	0.9817
TAR_180 & Grain products	-0.3	0.0738	0.6396
TAR_180 & Fats and oils	-0.06	0.7195	0.9354
TAR_180 & Fruits	-0.35	0.0313	0.5993
TAR_180 & Vegetables	-0.15	0.3643	0.7592
TAR_180 & Meat and fish products	-0.08	0.6446	0.8821
TAR_180 & Beverages	0.19	0.2651	0.7592
TAR_180 & Sweetened beverages	0.14	0.4088	0.7592
TAR_180 & Simple sugars	-0.22	0.1822	0.7592
TAR_180 & Simple sugars (excluding fruits)	-0.01	0.9506	0.9817
TAR_180 & PAQ - spare time activity	0.04	0.7916	0.9801
TAR_180 & PAQ - overall activity	0	0.9817	0.9817
TAR_180 & Ankieta_sumarycznie	-0.01	0.9592	0.9817
TAR_180 & PedsQL Diabetes	0.1	0.5576	0.8278
TAR_180 & PedsQL Treatment I	0.11	0.5282	0.8278
TAR_180 & PedsQL Treatment II	0.1	0.5731	0.8278
TAR_180 & PedsQL Worries	0.15	0.3885	0.7592
TAR_180 & PedsQL Communication	0.19	0.2507	0.7592
TAR_180 & PedsQL Total	0.17	0.3078	0.7592

DDI – Daily Dose of Insulin, GMI – Glucose Management Indicator, CV – Coefficient of Variation, TBR70 – Time Below Range <70 mg/dL, TITR – Time In Tight Range, TIR – Time In Range, TAR180 – Time Above Range >180 mg/dL, PAQ – Physical Activity Questionnaire, PedsQL – Pediatric Quality of Life Inventory.
